# Increased hip fracture risk in the patients with type 2 diabetes mellitus is correlated with urine albumin-to-creatinine ratio (ACR) and diabetes duration in men

**DOI:** 10.3389/ebm.2024.10240

**Published:** 2024-12-13

**Authors:** Huiru Ding, Hongxia Wang, Guanghui Liu, Yu Wang, Dongxu Han, Xiaoya Zhang, Lige Song

**Affiliations:** ^1^ Department of Endocrinology, Shanghai Tongji Hospital, School of Medicine, Tongji University, Shanghai, China; ^2^ Institute of Osteoporosis and Metabolic Bone Diseases, School of Medicine, Tongji University, Shanghai, China

**Keywords:** type 2 diabetes mellitus, fracture risk, hip fracture, urine albumin to creatinine ratio, diabetes duration

## Abstract

Patients with type 2 diabetes mellitus (T2DM) have increased hip fracture risk. And the association between urine albumin to creatinine ratio (ACR) and an increased risk of hip fracture in patients with T2DM remains controversial. This study aimed to investigate the association between urinary ACR and hip fracture risk in postmenopausal women and aged men with T2DM. The study included 219 postmenopausal women and 216 older men (mean age >60 years) with T2DM. Women and men were divided into control group (ACR<30 mg/g), microalbuminuria group (30 mg/g ≤ ACR<300 mg/g), and macroalbuminuria group (ACR≥300 mg/g) respectively. Demographic characteristics and clinical history were collected in patients. Biochemical indexes and bone turnover-related markers were measured in patients. In the study, we found that several factors, including age, T2DM duration, cerebral infarction history, serum corrected calcium levels and urine ACR were positively associated with hip fracture risk. However, 25-Hydroxyvitamin D and areal BMD were negatively associated with hip fracture risk. Furthermore, multiple regression analysis showed that urinary ACR level (β = 0.003, p = 0.044) and duration of T2DM (β = 0.015, p = 0.018) were positively and independently correlated with hip fracture risk in older men. In contrast, femoral neck BMD (β = −6.765, p < 0.001) was independently and negatively correlated with hip fracture risk in older men. This study indicated that the elevated ACR levels and longer T2DM duration were related to higher hip fracture risk in older men with T2DM, which could be beneficial for developing a predictive model for osteoporotic fractures in patients with type 2 diabetes in the future. However, results were inconsistent in women, hip fracture risk didn’t alter by changes in urinary microalbuminuria level in postmenopausal women with T2DM.

## Impact statement

1. Hip fracture was related to age, T2DM duration, cerebral infarction history, serum corrected calcium levels, 25-Hydroxyvitamin D, urine ACR and areal BMDs. 2. Urinary ACR level and duration of T2DM were independent predictors of hip fracture risk in older men by multiple linear regression analysis incorporating these correlates. 3. Increased hip fracture risk in older men with T2DM was related to elevated ACR levels and longer T2DM duration.

## Introduction

Diabetes mellitus (DM) is a common metabolic disease with increasing prevalence throughout the world. Chronic complications of DM adversely affect multiple organ systems, which cause an enormous medical and economic burden and reduce life span [1]. Apart from the significant complications, such as diabetic nephropathy, diabetic retinopathy and diabetic peripheral neuropathy, diabetic osteopathy is also reported as a severe complication for heightened risk of fractures among diabetic patients [2]. In type 1 diabetes (T1DM), reduced bone mass and bone strength, increase the susceptibility to fractures. In contrast, Type 2 diabetes mellitus also have been reported to be associated with an increased fractures risk, vertebral [3], hip [4] and all non-vertebral fractures. Previous studies have demonstrated that many factors contribute to the increased risk of fractures in type 2 diabetes mellitus, including impaired insulin signaling, decreased incretin effect, increased oxidative stress, accumulation of advanced glycation end products, and microvascular damage [5]. Diabetic nephropathy is recognized as one of the most important microvascular complications of diabetes, which has been reported to increase the risk of fracture in patients with T2DM [6].

Studies confirmed disturbances in serum calcium, phosphate and vitamin D levels, parathyroid hormone (PTH) metabolism, and dysregulation of bone turnover, primarily attributed to progressive eGFR decline associated with diabetic nephropathy [7, 8]. Besides, it is worth noting that persistent albuminuria is a marker of microvascular injury in diabetic nephropathy. Previous studies have demonstrated that microcirculation is vital to bone health [9]. And several clinical studies have demonstrated high urinary ACR levels maybe associated with increased fracture risk in non-diabetic populations [8, 10, 11]. However, patients with T2DM have unique skeletal metabolic features, and the association between ACR and fracture risk among the individuals with T2DM is unclear. Our study mainly explored whether urinary albumin excretion can be used as a risk factor for fractures in patients with type 2 diabetes.

## Materials and methods

### Study population

This was a cross-sectional study. It enrolled 435 individuals with type 2 diabetes mellitus who came to the Department of Endocrinology of Shanghai Tongji hospital for regular follow-up from January 2019 to December 2020. The enrolled population consisted of 219 post-menopausal women (menopausal status was confirmed by the absence of menses for more than 1 year in a woman over 50 years of age; and, in women with previous hysterectomy or those under 50 years of age, by an elevated value for serum follicle-stimulating hormone (FSH) of >30 IU/L) between 50 and 90 years old and 216 men between 40 and 80 years old. All patients were diagnosed with T2DM based on the criteria of the American Diabetes Association [12].

All patients met the following criteria: (1) all women were post-menopausal, and all men were over 40 years old; (2) the eGFR was greater than 30 mL/min/1.73 m^2^ in all patients; and (3) never use any anti-osteoporosis drugs. The exclusion criteria included: (1) severe hepatic dysfunction with alanine aminotransferase >40 U/L or aspartate aminotransferase >40 U/L; (2) severe renal dysfunction with eGFR <30 mL/min/1.73 m^2^; (3) malignancy; (4) hyperthyroidism; (5) rheumatoid arthritis; (6) hormone replacement therapy for the last 6 months; (7) prior anti-osteoporotic medicine; (8) previous use of a hypoglycemic agent that can affect bone metabolism such as thiazolidinediones (TZDs); and (9) depression diseases. At last, we selected 435 patients with T2DM who met the above criteria. The study has passed the Ethics Committee of Tongji Hospital and Tongji University School of Medicine and conforms to the Declaration of Helsinki. All participants signed informed consent. This study has been registered in the Chinese Clinical Trials Registry (ID: ChiCTR1800020077).

### Demographic information and clinical history

Each patient’s medical history was reviewed. The following data were recorded: age, sex, height, weight, current smoking and drinking status (there are many people who drink but who are not consuming excessive amounts of alcohol. Therefore drinking status referes to excessive alcohol consumption, which is defined as drinking alcohol 3 or more units per day.), duration of type 2 diabetes, history of coronary heart disease, hypertension, and cerebral infarction. Body mass index (BMI) (kg/m^2^) was calculated as weight (kg)/height (m^2^). Drinking status was defined as consuming three or more units of alcohol daily (one unit of alcohol means 8 g of alcohol).

### Laboratory measurements

Peripheral venous blood was collected after overnight fast to measure biochemical data, including fasting blood glucose (FBG), fasting insulin, glycosylated haemoglobin (HbA1c), liver and renal function, serum albumin level, lipid profile including total cholesterol (TC), triglyceride (TG), low-density lipoprotein (LDL) and high-density lipoprotein (HDL), electrolyte levels including serum calcium (Ca) and phosphorus (P), bone turnover markers, and bone metabolism-related hormones including bone alkaline phosphatase (BALP), procollagen type I intact N- terminal (P1NP), osteocalcin (OC), tartrate-resistant acid phosphatase-5b (TRACP-5b), C- terminal cross-linking telopeptide of type I collagen (CTX), 25-hydroxy vitamin (D [25(OH)D]), and parathyroid hormone (PTH).

HbA1c was detected using high-performance liquid chromatography with whole blood. Serum OC, CTX, P1NP, [25-(OH)D], and PTH were measured by electrochemiluminescence assay (Roche Diagnostics; coefficient of variation of intra- and inter-assay <10%). The enzyme immunoassay was used to measure the serum BALP and TRACP- 5b levels (IDS Ltd; coefficient of variation of intra- and inter-assay <10%). An automatic chemistry analyzer using serum detected liver function, renal function, albumin, lipid profiles, and electrolytes. Corrected Ca (mmol/L) was calculated according to the following formula: Ca (mmol/L) −0.025*albumin+1. The calcium-phosphorus product was calculated using the following formula: 12.4*Ca (mmol/L) *P (mmol/L). The insulin resistance (homeostatic model assessment for insulin resistance, HOMA-IR) assessment was calculated according to the existing literature formula: FBG (mmol/L) *fasting insulin (mU/L)/22.5 [13]. The arterial stiffness index or the atherogenic coefficient was calculated as follows: (TC-HDL-C)/HDL-C [14–16].

The eGFRs (ml/min/1.73 m2) were estimated using the serum creatinine (sCr) levels according to the simplified CKD-MDRD equation as follows [17].

$$\text{For male}:\text{eGFR}=186*\text{sCr}-1.154*{\text{age}}^{-0.203}$$

$$\text{For female}:\text{eGFR}=\left(186*\text{sCr}-1.154*{\text{age}}^{-0.203}\right)*0.742$$

Fasting urine samples were also collected to measure urine albumin and urine creatinine levels using an immunoturbidimetric method. The urine albumin/creatinine ratios (mg/g) were calculated by dividing the urine microalbumin by the urine creatinine concentration. The sensitivity and precision of urine albumin are 93% and 94%, and the sensitivity and precision of urine creatinine are 91% and 97%. According to clinical recommendations from the American Diabetes Association (ADA) for the prevention and control of diabetic nephropathy, study participants were categorized into three groups based on ACR: the normal group (ACR<30 mg/g), the microalbuminuria group (30 mg/g ≤ ACR≤300 mg/g), and macroalbuminuria group (ACR >300 mg/g). Among women, there were 20 patients in the macroalbuminuria group, 79 patients in the microalbuminuria group, and 120 patients in the control group. Among men, there were 33, 45, and 138 patients in the respective groups.

### BMD and fracture risk

Bone mineral density (BMD) at lumbar spine 1–4, femoral neck, and total hip was measured using dual-energy X-ray absorptiometry (DXA) at Shanghai Tongji Hospital with a Hologic instrument (Hologic Inc., United States). The coefficient of variation was less than 1.0%. Least significant change (LSC) was 2.31%. The 10-year probability of significant osteoporotic fractures (including the clinical spine, hip, humerus, or wrist) was predicted using the FRAX tool and accessed through the International Osteoporosis Foundation (IOF) website^1^. Fracture risk was calculated based on age, BMI, femoral neck BMD, and dichotomized risk factors [18].

### Statistical analysis

We used SPSS version 26.0 (SPSS Inc., Chicago, IL, United States) to perform all statistical-analyses. All continuous variables were analyzed using the Kolmogorov -Smirnov test to test normality of data. Non-normal data generally exhibit a normal distribution after logarithmic transformation. Normally distributed continuous variables were described as mean ± SD. Moreover, the median (p25-p75) was used to describe skewness distribution variables. These skewness distribution variables were transformed using a logarithmic transformation to make them normally distributed and render them suitable for analysis of variance (ANOVA). Categorical variables were reported as numbers and percentages. One-way ANOVA was applied to compare the baseline data among the three groups for continuous variables. The least significant difference (LSD) method was used to compare baseline data between the two groups.

Furthermore, the chi-square test was used to compare the categorical variables. The Pearson correlation was conducted to analyze whether partial discrepant variables were associated with hip fractures in males. Multiple regression analysis was used to assess the independent associations between various clinical factors and the risk of hip fracture. We included variables that showed potential associations (p < 0.1 according to Pearson correlation) in the multiple linear regression model. Values of p less than 0.05 were considered statistically significant.

## Results

### Comparison of characteristics of patients in the control, microalbuminuria, and macroalbuminuria groups

Table 1 showed the clinical characteristics of 219 post-menopausal women and 216 men. The average age of all patients was over 65 years old, and there were no differences observed among the three groups of females. In men, the mean age of the microalbuminuria group (70.47 ± 8.28) was higher than that of the control group (63.55 ± 9.85) and the macroalbuminuria group (64.61 ± 9.87) (p < 0.001), while there was no significant difference between the control and macroalbuminuria groups. In addition, at baseline, the duration of diabetes, homeostatic model assessment for insulin resistance (HOMA-IR), serum corrected calcium level, and serum total alkaline phosphatase (ALP) level were significantly different among three groups both in women and men (all p < 0.05; Table 1). In women, the microalbuminuria group had longer T2DM duration [16.0 (9.0–20.0), p = 0.029] and HOMA-IR [6.0 (3.40–11.62), 0.021] than control group. For men, the macroalbuminuria group had longer T2DM duration [15.0 (6.5–20.0), p = 0.010] than control group. Regardless of sex, individuals with T2DM with microalbuminuria had higher total ALP levels [women: 83.85 (74.33–99.38) U/L, p = 0.024; 95.15 (76.30–109.75)U/L, P = 0.023] than control groups. However, the macroalbuminuria group of women had higher corrected Ca [2.41 (2.26–2.45) mmol/L, p = 0.012] level than control group and the microalbuminuria group of men had had higher corrected Ca [2.34 (2.24–2.35) mmol/L, p = 0.005] level than control group. The significant differences in atherogenic coefficient, glycosylated haemoglobin and serum creatinine levels were found among the three groups of post-menopausal women (all p < 0.05; Table 1), no finding in men. In women, the macroalbuminuria group had higher atherogenic coefficient (4.013 ± 1.166, p = 0.011) than other two groups, the microalbuminuria group had higher glycosylated haemoglobin level [9.6%, (8.30%–11.40%) p = 0.023] than control group and the macroalbuminuria group had higher serum creatinine level [75.30 (62.50–94.15) µmol/L, p = 0.015) than control group. Intergroup differences in hypertension, history of cerebral infarction, serum 25(OH)D level, total hip BMD measurements, and hip fracture were observed in men but not in women (Table 1). In men, the microalbuminuria group had lower total hip BMD (0.841 ± 0.150 g/cm^2^, p = 0.035), higher history of cerebral infarction prevalence (71.1%, p < 0.001) and a higher hip fracture risk [0.90 (0.60–1.55), p = 0.014] compared to the other tow groups. In both women and men, there were no significant differences observed in other indices among the control, microalbuminuria, and macroalbuminuria groups (all p > 0.05; Table 1).

**TABLE 1:** Comparison of characteristics of patients in the normal, microalbuminuria, and macroalbuminuria groups.

Factors	Sex = Female (N = 219)			
	Control (N = 120)	Microalbuminuria (N = 79)	Macroalbuminuria (N = 20)	P-value
Demographic and history				
Age (years)	66.97 ± 9.33	68.84 ± 9.99	67.15 ± 7.53	0.382
BMI (kg/m2)	24.535 ± 3.214	24.721 ± 3.371	24.293 ± 2.452	0.777
Current smoking (%)	4 (3.3%)	3 (3.8%)	0 (0)	0.684
Current drinking (%)	0 (0%)	0 (0%)	0 (0%)	—
DM Duration (years)	11.0 (7.7–20.0)	16.0 (9.0–20.0)^a^	14.0 (10.25–20.0)	0.029^*^
History of coronary heart disease	29 (24.2%)	30 (38%)	8 (40%)	0.074
History of hypertension	71 (59.2%)	57 (72.1%)	15 (75%)	0.108
History of cerebral infarction	31 (25.8%)	26 (32.9%)	5 (25%)	0.523
Blood biochemical indicators				
HbA1c (%)	8.60 (7.40–10.65)	9.60 (8.30–11.40)^a^	9.30 (7.28–10.70)	0.023^*^
HOMA-IR	4.46 (2.39–7.59)	6.00 (3.40–11.62)^a^	5.56 (3.19–8.70)	0.021^*^
TCH (mmol/L)	4.95 ± 1.29	4.93 ± 1.37	5.48 ± 1.95	0.247
TG (mmol/L)	1.61 (1.12–2.11)	1.84 (1.31–2.33)	1.84 (1.38–2.99)	0.064
LDL (mmol/L)	3.34 ± 1.03	3.27 ± 0.98	3.67 ± 1.23	0.284
HDL (mmol/L)	1.14 (1.00–1.36)	1.11 (0.96–1.27)	1.08 (0.92–1.28)	0.140
Atherogenic coefficient	3.241 ± 1.042	3.356 ± 1.038	4.013 ± 1.166^a^ ^b^	0.011^*^
Corrected Ca (mmol/L)	2.29 (2.23–2.35)	2.31 (2.26–2.39)	2.41 (2.26–2.45)^a^	0.012^*^
P (mmol/L)	1.27 ± 1.18	1.27 ± 0.20	1.30 ± 0.23	0.867
The calcium-phosphorus product	35.45 (32.42–40.13)	36.07 (32.79–39.93)	37.15 (31.46–42.56)	0.303
sCr (umol/L)	66.65 (59.90–79.35)	79.00 (61.40–90.60)^a^	75.30 (62.50–94.15)^a^	0.015^*^
eGFR (ml/min/1.73 m^2^)	79.201 ± 20.941	75.042 ± 24.726	67.666 ± 29.823	0.093
Bone metabolism-related markers				
Total ALP(U/L)	76.00 (62.25–92.78)	83.85 (74.33–99.38)^a^	80.00 (63.00–94.05)	0.024
BALP (ug/L)	14.03 (10.70–17.51)	14.24 (10.80–19.07)	15.05 (11.00–19.80)	0.579
P1NP(ng/mL)	38.05 (31.48–49.30)	36.95 (33.05–42.00)	44.65 (28.55–50.25)	0.820
OC(ng/mL)	12.15 (8.53–14.67)	11.20 (8.71–14.40)	13.81 (11.03–17.91)	0.097
TRACP-5b (U/L)	0.964 ± 0.338	0.959 ± 0.322	0.907 ± 0.342	0.776
CTX (ng/mL)	0.373 (0.245–0.483)	0.359 (0.245–0.530)	0.429 (0.254–0.709)	0.657
25(OH)D (nmol/L)	40.60 (32.77–53.99)	39.67 (34.31–51.45)	34.62 (25.87–47.79)	0.205
PTH(pg/mL)	33.780 (15.07)	32.175 (22.96)	42.70 (42.07)	0.139
Bone mineral density by DXA				
Lumbar1-4 BMD (g/cm^2^)	0.875 (0.772–0.964)	0.886 (0.799–0.979)	0.852 (0.761–0.987)	0.663
Femur neck BMD (g/cm^2^)	0.645 ± 0.116	0.620 ± 0.120	0.605 ± 0.139	0.188
Total hip BMD (g/cm^2^)	0.806 ± 0.123	0.773 ± 0.129	0.775 ± 0.126	0.160
Fracture risk by FRAX tool				
Major osteoporotic fracture	3.90 (3.08–5.90)	4.50 (3.50–5.85)	5.30 (2.80–6.50)	0.327
Hip Fracture	1.00 (0.48–2.33)	1.40 (0.60–2.50)	1.90 (0.33–2.30)	0.123

Factors	Sex = Male (N = 216)			
	Control (N = 138)	Microalbuminuria (N = 45)	Macroalbuminuria (N = 33)	P-value
Demographic and history				
Age (years)	63.55 ± 9.85	70.47 ± 8.28^a^	64.61 ± 9.87^b^	0.000^***^
BMI (kg/m2)	24.293 ± 3.083	23.655 ± 2.915	25.069 ± 3.152	0.133
Current smoking (%)	51 (37%)	15 (33.3%)	15 (45.5%)	0.538
Current drinking (%)	22 (15.9%)	8 (17.8%)	5 (15.2%)	0.944
DM Duration (years)	9.0 (5.0–15.0)	10.0 (5.5–20.0)	15.0 (6.5–20.0)^a^	0.010^**^
History of coronary heart disease	37 (26.8%)	12 (26.7%)	15 (45.5%)	0.096
History of hypertension	74 (53.6%)	32 (71.1%)	32 (97%)^a^ ^b^	0.000^***^
History of cerebral infarction	32 (23.2%)	23 (51.5%)^a^	9 (27.3%)	0.002^**^
Blood biochemical indicators				
HbA1c (%)	8.70 (7.20–13.00)	10.05 (8.45–11.50)	9.30 (7.65–11.35)	0.079
HOMA-IR	4.03 (2.22–6.92)	5.59 (3.38–10.24)^a^	6.16 (2.65–9.12)	0.005^**^
TCH (mmol/L)	4.37 ± 1.04	4.16 ± 0.91	4.58 ± 1.29	0.315
TG (mmol/L)	1.39 (0.97–1.98)	1.31 (1.00–1.85)	1.45 (1.12–2.44)	0.218
LDL (mmol/L)	2.94 ± 0.83	2.78 ± 0.72	2.98 ± 0.91	0.491
HDL (mmol/L)	1.00 (0.86–1.16)	0.98 (0.84–1.11)	1.00 (0.82–1.15)	0.975
Atherogenic coefficient	3.417 ± 1.192	3.228 ± 1.071	3.631 ± 1.218	0.324
Corrected Ca (mmol/L)	2.26 (2.22–2.33)	2.34 (2.24–2.35)^a^	2.32 (2.27–2.37)	0.005^**^
P (mmol/L)	1.19 ± 0.18	1.15 ± 0.19	1.19 ± 0.22	0.511
The calcium-phosphorus product	33.55 (28.98–37.13)	32.86 (29.05–38.22)	32.86 (29.18–38.04)	0.814
sCr (umol/L)	84.20 (74.00–96.30)	85.00 (73.25–101.75)	89.90 (75.50–126.00)	0.096
eGFR (ml/min/1.73m2)	85.262 ± 20.868	81.789 ± 24.546	78.958 ± 26.756	0.300
Bone metabolism-related markers				
Total ALP(U/L)	76.20 (66.60–90.00)	95.15 (76.30–109.75)^a^	84.40 (64.50–97.35)	0.023
BALP (ug/L)	12.70 (10.55–18.23)	14.79 (10.02–17.30)	12.41 (10.51–16.05)	0.444
P1NP(ng/ml)	36.40 (30.20–42.63)	37.80 (30.90–43.40)	31.90 (29.00–38.95)	0.207
OC(ng/ml)	9.71 (8.23–12.70)	9.70 (8.01–12.32)	10.00 (6.96–12.39)	0.440
TRACP-5b (U/L)	0.874 ± 0.311	0.933 ± 0.256	0.834 ± 0.363	0.349
CTX (ng/ml)	0.305 (0.223–0.476)	0.313 (0.232–0.418)	0.249 (0.190–0.369)	0.745
25(OH)D (nmol/L)	45.23 (34.62–57.09)	40.07 (28.93–46.11)^a^	30.15 (20.60–46.69)^a^ ^b^	0.000^***^
PTH(pg/ml)	40.351 ± 18.555	34.645 ± 13.559	35.962 ± 17.073	0.110
Bone mineral density by DXA				
Lumbar1-4 BMD (g/cm^2^)	0.960 (0.885–1.095)	0.991 (0.906–1.095)	1.038 (0.878–1.199)	0.430
Femur neck BMD (g/cm^2^)	0.728 ± 0.117	0.681 ± 0.117	0.714 ± 0.126	0.071
Total hip BMD (g/cm^2^)	0.900 ± 0.133	0.841 ± 0.150^a^	0.901 ± 0.125	0.035^*^
Fracture risk by FRAX tool				
Major osteoporotic fracture	2.25 (1.70–2.90)	3.25 (2.50–4.32)	2.40 (1.80–2.98)	0.105
Hip Fracture	0.60 (0.30–1.13)	0.90 (0.60–1.55)^a^	0.70 (0.30–1.50)	0.014^*^

Values are shown as means ± SD, median (p25-p75), or number (percentage).

^a^ p < 0.05 compared with the normal group.

^b^ p < 0.05 compared with the microalbuminuria group.

Abbreviations: BMI, body mass index; DM, diabetes mellitus; HbA1c, glycosylated hemoglobin; HOMA-IR, homeostatic model assessment for insulin resistance; HDL, high density lipoprotein; LDL, low density lipoprotein; TCH, total cholesterol; TG, triglyceride; corrected Ca, corrected calcium; P, phosphorus; sCr, serum creatinine; eGFR, glomerular filtration rate; Total ALP, total alkaline phosphatase; BALP, bone alkaline phosphatase; OC, osteocalcin; TRACP-5b, tartrate-resistant acid phosphatase-5b; CTX, C-terminal cross-linking telopeptide of type I collagen; P1NP, procollagen 1 intact N-terminal; 25(OH)D, 25-hydroxyvitamin D; PTH, parathyroid hormone; DXA, dual-energy X-ray absorptiometry; BMD: bone mineral density. *p < .05, **p < .01, ***p< 0.001.

### Correlation analysis of clinical characteristics with hip fracture in men

Bivariate correlation analysis showed that several clinical characteristics in men with type 2 diabetes mellitus, including age (r = 0.463, p < 0.001), duration of type 2 diabetes mellitus (r = 0.154, p = 0.023), history of cerebral infarction (r = 0.223, p = 0.050), serum corrected calcium level (r = 0.140, p = 0.040), and ACR (r = 0.176, p = 0.010) were positively related to hip fracture. Bone mineral density (BMD) measurements at the lumbar vertebrae (r = −0.428, p < 0.001), femoral neck (r = −0.909, p < 0.001) and total hip (r = −0.720, p < 0.001), as well as with serum 25(OH)D levels (r = −0.134, p = 0.050) showed negative association with hip fractures. However, history of hypertension, HOMA-IR, and serum total ALP level were not significantly correlated with the total hip fracture in men (p > 0.05) (Table 2).

**TABLE 2:** Correlation analysis of different characteristics with Hip Fracture in male.

Indexes	r	P
Age	0.463	<0.001^***^
DM Duration	0.154	0.023^*^
History of hypertension	0.043	0.526
History of cerebral infarction	0.223	0.001^***^
Corrected Ca	0.140	0.040^*^
HOMA-IR	−0.067	0.325
Total ALP	0.064	0.351
25(OH)D	−0.134	0.050^*^
ACR	0.176	0.010^**^
Lumbar 1-4 BMD	−0.428	<0.001^***^
Femur neck BMD	−0.909	<0.001^***^
Total hip BMD	−0.720	<0.001^***^

Abbreviations: DM, diabetes mellitus; corrected Ca, corrected calcium; P, phosphorus; sCr, serum creatinine; eGFR, glomerular filtration rate; Total ALP, total alkaline phosphatase; 25(OH)D, 25-hydroxyvitamin D; ACR, albumin/creatinine ratio; BMD, bone mineral density. *p < .05, **p < .01, ***p < 0.001.

### Multiple linear regression analysis of hip fracture in men

For men with type 2 diabetes mellitus, multiple linear regression analysis was performed to identify independent influence factors of hip fracture. Femoral neck BMD was negatively correlated with hip fracture risk (β = −6.765, p < 0.001) (Table 3). In contrast, T2DM duration (β = 0.015, p = 0.018) and ACR (β = 0.003, p = 0.044) were positively associated with hip fracture risk. And the results remained statistically significant after further adjustment by age, history of cerebral infarction, serum corrected Ca and 25(OH)D levels, urinary ACR level, and areal BMDs (Table 3).

**TABLE 3:** Multiple regression analysis of Hip Fracture in male.

Indexes	β	Lower limit of 95% CI	Upper limit of 95% CI	P-value
Age	0.009	−0.002	0.020	0.102
DM Duration	0.015	0.003	0.028	0.018^*^
History of cerebral infarction	0.021	−0.196	0.237	0.850
corrected Ca	0.104	−0.446	0.653	0.711
25(OH)D	−0.002	−0.007	0.004	0.509
ACR	0.003	0.000	0.006	0.044^*^
Lumbar 1-4 BMD	0.098	−0.625	0.821	0.790
Femur neck BMD	−6.765	−8.247	−5.283	<0.001^***^
Total hip BMD	0.336	−0.997	1.669	0.620

Abbreviations: DM, diabetes mellitus; corrected Ca, corrected calcium; 25(OH)D, 25-hydroxyvitamin D; ACR, albumin/creatinine ratio; BMD, bone mineral density. *p < .05, ***p < 0.001.

Results were inconsistent in women, hip fracture risk didn’t alter by changes in urinary microalbuminuria level in postmenopausal women with T2DM.

## Discussion

Type 2 diabetes mellitus has been reported to be associated with an increased risk of fracture [19–21], and the increased hip fracture risk appears to be the most obvious [19]. However, the underlying causes and risk factors still require further investigation. Several large clinical studies have found an increased fracture risk among individuals with T2DM. One Women’s Health Initiative Observational Study (WHI-OS), involving 93,676 postmenopausal women with T2DM, adjusted for frequent falls and increased areal BMDs, revealing heightened risk of hip, foot, and spine fractures [22]. Secondly, conducted in 5994 men (≥65 years), found that non-vertebral fracture risk was higher in patients with diabetes who were using insulin compared with non-diabetic patients (HR1.74; 95%CI:1.31–2.69) [23]. In addition, in a recent meta-analysis of 15 studies (n = 852705), people with type 2 diabetes had a 35% higher incidence of fracture [24].

One of the goals of this cross-sectional study was to investigate the relationship between urinary ACR levels and fracture risk among elderly patients with T2DM, who had an eGFR greater than 30 mL/min/1.73 m^2^ and average serum creatine levels less than 100 mmol/L. We found that urinary ACR levels were associated with a slight but statistically significant increase in hip fracture risk among men with T2DM. Specifically, hip fracture risk appeared higher in men with ACR levels equal to or greater than 30 mg/g but less than 300 mg/g compared to those with levels below 30 mg/g. However, so few men had macroalbuminuria that we found no difference in the risk of hip fracture between the group with macroalbuminuric group and the other two groups in our study.

The urinary albumin-to-creatinine ratio (ACR) reflects the status of diabetic microangiopathy [25]. Microcirculation significantly influences bone health [26, 27]. Previous large clinical studies have shown a correlation between urinary ACR levels and fracture risk. A mean follow-up of 4.6 years from the ONTARGET and TRANSCEND trials found that baseline albuminuria levels were associated with increased risk of hip and pelvic fractures. Importantly, this association was consistent across sexes and between diabetic and non-diabetic populations [28]. Barzilay et al. found that urinary microalbumin levels were linked to an increased risk of hip fractures in older women, but not in men [29]. A prospective study of community-dwelling older men aged ≥65 years found no independent association of urine albumin with the risk of incident fracture [10]. The risk estimates from our current study are not fully consistent with previous studies. The current study focused on individuals with type 2 diabetes, whose distinctive metabolic characteristics may alter fracture susceptibility compared to older adults without type 2 diabetes. Despite including a limited number of patients with macroalbuminuria, we still found a dose-dependent relationship between urinary ACR levels and the risk of hip fractures, thereby substantiating our finding. However, the effect of macroalbuminuria ACR levels on hip fracture risk observed in this study was too small to be used for clinical prognostic purposes. Further research may explore its potential role in larger cohorts populations.

The impact of urinary ACR levels on fracture risk in patients with type 2 diabetes can be attributed to several key factors. Firstly, inadequate blood flow in the bone microvasculature can lead to increased cortical porosity. This change in bone microarchitecture results in reduced bone strength and an increased likelihood of fractures [30, 31]. Secondly, when microalbuminuria occurs, levels of inflammatory cytokines are typically elevated [32]. While elevated levels of inflammatory cytokines are associated with osteoporosis [33], which indirectly increases the risk of fracture. Thirdly, microangiopathy contributes to muscle and nerve damage, thereby increasing the risk of falls.

At the same time, the conclusion of our study corroborates previous findings indicating that the duration of type 2 diabetes independently contributes to the fracture risk of patients with T2DM [6, 34, 35]. A prospective study involving 3,654 subjects aged 49 years and older found that longer duration of diabetes is associated with an increased fracture risk [36]. Another retrospective, population-based matched cohort study spanning from 1984 to 2004 found that long-term diabetes is associated with an increased fracture risk, whereas newly diagnosed diabetes is associated with a reduced fracture risk [37]. The reason why the duration of type 2 diabetes remains associated with fracture risk after adjusting for effects such as bone density may be multifactorial. Firstly, patients with longer disease duration tend to have more comorbidities and poorer nutritional status, including decreased muscle strength and muscle mass, leading to an increased risk of falls [38]. Secondly, as the disease progresses, there is an increase in advanced glycation end products in bones, leading to decreased bone strength.

However, we didn’t observe the similar results in post-menopausal women. Hip fracture risk didn’t alter by changes in urinary microalbuminuria level in postmenopausal women with T2DM. This may be attributed to the following factors. Firstly, there is a significant disparity in fracture risk between men and women. Previous studies have shown that osteoporotic or fragility fractures affect one in two women and one in five men who are older than 50 [39]. Men usually have a higher peak bone mass compared to women. This means that, all else being equal, men start with a stronger skeletal structure, reducing their fracture risk. Women, especially postmenopausal women, experience a rapid decline in bone density due to hormonal changes (mainly the decrease in estrogen levels), which makes them more prone to osteoporosis and fractures. In addition to lower bone mineral density, women, especially older women, are more susceptible to falls due to decreased muscle strength, balance issues and other factors like a higher body fat percentage, all of which increase their risk of fractures [40]. This rationale also underpinned the separate analysis of fracture risk for men and women in this study. Secondly, the effect of ACR on fracture risk in women is diminished by other key factors, such as the post-menopausal decline in bone mineral density due to reduced estrogen levels, as well as the heightened fracture risk linked to decreased muscle mass. Thus, future studies with larger sample sizes and more rigorous methodologies should aim to incorporate as many relevant fracture risk factors as possible to more accurately assess their individual contributions and facilitate the development of a more comprehensive fracture risk assessment model.

In addition, we observed the HbA1c and HOMA-IR levels were high. HOMA-IR in the controls was >4 and even higher in the other groups. While HbA1c% was >8.6 in all groups. These findings suggested that patients didn’t achieve optimal diabetes management, placing them at an increased risk of both non-fatal and fatal cardiovascular events. This could be attributed to that this study included patients who visited Tongji Hospital in Shanghai. They typically sought medical care due to symptoms related to poor blood glucose control. Consequently, we observed that patients generally had suboptimal blood glucose levels. Moreover, recent research indicates that achieving a single blood glucose control target is no longer the sole goal in managing diabetes. Preventing non-fatal and fatal cardiovascular events is a key goal in managing type 2 diabetes patients [41]. In recent years, there has also been increasing concern about the high risk of fractures in diabetic patients. Therefore, reducing the risk of fractures in diabetic patients is also becoming one of the management goals for diabetes.

Here are several strengths of this study: (1) We analyzed bone metabolism and fracture risk separately for men and women to rule out the influence of gender differences; (2) Cases with eGFR>30 mL/min/1.73 m^2^ were selected; (3) Study participants were categorized into three groups based on ACR: the normal group (ACR<30 mg/g), the microalbuminuria group (30 mg/g ≤ ACR≤300 mg/g), and macroalbuminuria group (ACR >300 mg/g), which may well reflect the severity of diabetic nephropathy; (4) BMD is a crucial factor determining bone strength and fracture susceptibility, but most brittle fractures occur in individuals without osteoporosis. Therefore, we chose the FRAX tool to calculate the probability better.

There are limitations to this study: (1) We have taken too few cases, especially people with T2DM with macroalbuminuria; (2) The medical history may be incomplete and associated with other undetected diseases; (3) Brittle fractures were not documented; (4) Urine ACR was grouped according to a single measurement. An abnormal level of ACR was confirmed with at least one further measurement because ACR has high within-person variability from day to day [42, 43]. (5) Fracture risk assessment tool (FRAX) widely used in the past underestimates fracture risk in people with T2DM [44], so our analyses may underestimate the impact of urinary ACR level on fracture. (6) Whether urine ACR directly affects bone microarchitecture is still unknown.

## Conclusion

In conclusion, we found that increased urinary ACR level and duration of T2DM were associated with an increased hip fracture risk among older men with T2DM. In addition, there was no difference between BMD and fracture risk among the three groups by urinary ACR level in postmenopausal women. In the future, it may be possible to reduce fracture risk in patients with T2DM by delaying the process of albuminuria and avoiding factors that increase fracture risk.

## Data Availability

The original contributions presented in the study are included in the article/supplementary material, further inquiries can be directed to the corresponding author.

## Footnotes

1
http://www.shef.ac.uk/FRAX/
